# Small Mammals as Carriers/Hosts of *Leptospira spp*. in the Western Amazon Forest

**DOI:** 10.3389/fvets.2020.569004

**Published:** 2020-12-02

**Authors:** Luciana dos Santos Medeiros, Susan Christina Braga Domingos, Maria Isabel Nogueira Di Azevedo, Rui Carlos Peruquetti, Narianne Ferreira de Albuquerque, Paulo Sérgio D'Andrea, André Luis de Moura Botelho, Charle Ferreira Crisóstomo, Anahi Souto Vieira, Gabriel Martins, Bernardo Rodrigues Teixeira, Filipe Anibal Carvalho-Costa, Walter Lilenbaum

**Affiliations:** ^1^Laboratório de Microbiologia e Imunologia Veterinária, Universidade Federal do Acre, Rio Branco, Brazil; ^2^Laboratório de Bacteriologia Veterinária, Universidade Federal Fluminense, Niterói, Brazil; ^3^Laboratório de Biologia e Parasitologia de Mamíferos Silvestres Reservatórios, IOC, Fiocruz, Rio de Janeiro, Brazil; ^4^Instituto Federal de Educação, Ciência e Tecnologia do Acre, Rio Branco, Brazil; ^5^Laboratório de Epidemiologia e Sistemática Molecular, IOC, Fiocruz, Rio de Janeiro, Brazil

**Keywords:** marsupial, small mammal, Amazon, wild rodent, sylvatic leptospirosis

## Abstract

*Leptospira* is a bacteria that causes leptospirosis and is transmitted through water, soil, or mud that is contaminated by the urine of infected animals. Although it is mainly associated with the urban environment, Leptospires also circulate in rural and wild environments. This study aimed to investigate the role of small mammals in leptospirosis epidemiology in the western Amazon, Brazil. In total, 103 animals from 23 species belonging to the orders Didelphimorphia and Rodentia were captured. Blood, kidney, and urine samples were collected and Microscopic Agglutination Test (MAT), *lip*L32 PCR, *sec*Y sequencing, and culturing were conducted. MAT was reactive on 1/15 sera, and no bacterial isolate was obtained. PCR yielded 44.7% positive samples from 16 species. Twenty samples were genetically characterized and identified as *L. interrogans* (*n* = 12), *L. noguchii* (*n* = 4), and *L. santarosai* (*n* = 4). No statistical association was found between the prevalence of infection by *Leptospira* spp. in small mammals within carrier/hosts species, orders, study area, and forest strata. Our results indicate a high prevalence of pathogenic *Leptospira* spp. in several rodent and marsupial species and report the first evidence of *Leptospira* spp. carrier/hosts in the Brazilian Western Amazon.

## Introduction

Leptospirosis is a zoonotic infectious disease caused by the bacteria from the *Leptospira* genus (1) and transmitted through water, soil, or mud contaminated by the urine of infected animals. Although it is mainly reported in the urban environment, leptospirosis also circulates in rural and wild environments, with a wide variety of mammal species acting as carrier/hosts of the bacterium (2).

The Amazonian biome has the ideal conditions for maintaining and disseminating *Leptospira* spp. The high humidity and temperature along with the high diversity of mammals and potential renal carriers of the bacteria, creates a scenario that exposes the animals to different strains of *Leptospira* (3, 4). Besides the importance of small mammals in the maintenance of *Leptospira* spp. in the wild and in the transmission to humans, the possibility of transmission between wild and domestic animals has been a major concern among conservationists and livestock authorities in many areas, especially in the Amazon (5–7). The emergence of zoonosis can greatly impact the abundance of some carrier/host populations, in some extreme cases leading to local extinctions (8).

Every mammal is a potential renal carrier of leptospires. Rodents have been described as the most essential maintenance/amplifier reservoirs in nature for several pathogenic *Leptospira* (9, 10). In Brazil, marsupials from the eastern Amazon (11), semi-arid regions (12), and the Atlantic Forest biome (13, 14) have also been reported with antibodies against *Leptospira* spp. The first report of *Leptospira* isolation in samples of *Didelphis albiventris* was from southern Brazil. These findings suggest marsupials may serve as important transmission reservoirs of pathogenic *Leptospira* spp. (15). Despite this, little is known about the role of wild small mammals as *Leptospira* carrier/hosts in the Amazon forest. Therefore, the objective of this study was to identify *Leptospira* infection in wild small mammals (rodents and marsupials) and investigate predictor variables related to infection in these animals in the Western Amazon, Brazil.

## Materials and Methods

### Study Design

**Figure 1:**
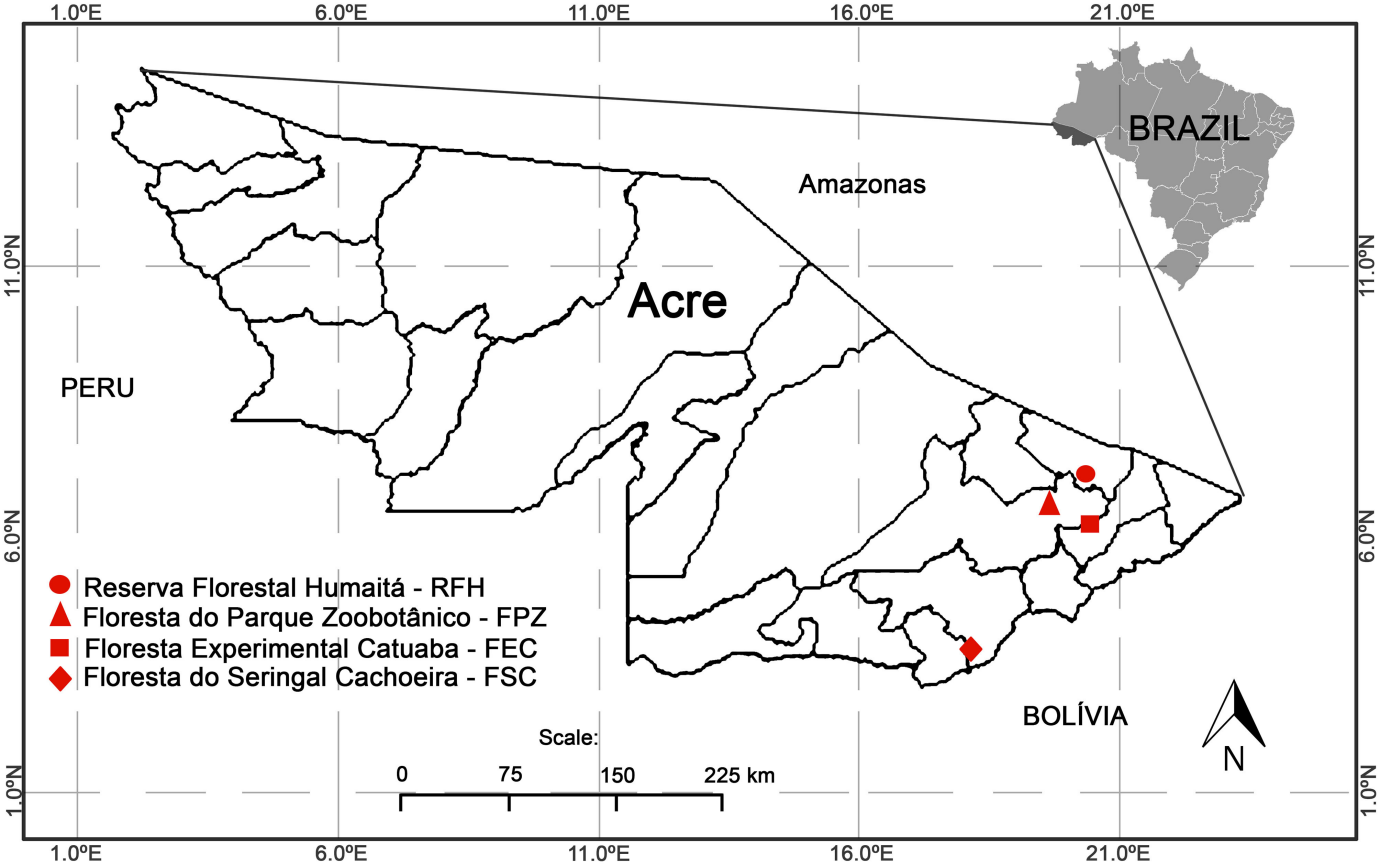
The four study areas located in the state of Acre. Floresta do Seringal Cachoeira (FSC): a continuous and conserved primary forest, Reserva Florestal Humaitá (RFH): a wide fragment of a primary rain forest, Floresta do Parque Zoobotânico (FPZ): an urban small fragment, and Floresta Experimental Catuaba (FEC): a secondary forest with agriculture.

The following licenses were used to conduct the study: permanent license to collect zoological material number 13373 (SISBIO-ICMBIO) and CEUA LW-39/14 license (Ethics Committee on Animal Use, FIOCRUZ). The studied areas were forests in the state of Acre, Brazil, western Amazon on Acre river basin, and represented different levels of conservation and land use (Figure 1). These areas included: (1) Floresta do Seringal Cachoeira (FSC) (10°49′S, 68°21′W), in the municipality of Xapuri, a continuous and conserved primary forest with 24,200 ha, a medium anthropic impact and land use for eco-tourism and traditional activities of latex and castanha-do-Brasil extractions; (2) Reserva Florestal Humaitá (RFH) (9°43′S, 67°48′W), in the municipality of Porto Acre, is a wide fragment of ~2,000 ha of primary and secondary rain forest, submitted to moderate anthropic action, surrounded by farms, roads and a large river. This area belongs to the Federal University of Acre (UFAC) and is a preserved area designated for research; (3) Floresta Experimental Catuaba (FEC) (10°04′S, 67°37′W), in the municipality of Senador Guiomard, comprising 900 ha of primary and secondary rain forest. This farm is used for research activities and also belongs to UFAC; and (4) Floresta do Parque Zoobotânico (FPZ) (9°57′S, 67°52′W), in the municipality of Rio Branco, an intense anthropized small fragment of approximately 140 ha in different succession stages. It is an urban park located on the UFAC campus, with land use destined to activities such as trekking, research, and recreation.

The field expeditions to capture small mammals and collect biological samples were conducted in the rainy seasons, between November and December 2015, at the RFH, FPZ, and FSC, and in FEC in December 2016.

### Animal Capture

Trapping was conducted over five consecutive nights. In each study area, the animals were captured with live traps, Sherman® (30 × 8 × 9 cm) and Tomahawk® (40 × 12 × 12 cm), in five to ten linear transects with 15 trapping stations each. Sampling was conducted at three forest strata—soil level, understory (at a height of 2 m), and canopy (15 m). At the end of each transect, four pitfalls were installed, with a volume of 60 L. The bait used was a mixture of bacon, oat, banana, and peanut butter. The captured animals were transported to a field laboratory base, where they were anesthetized and euthanized following the procedures reported previously (16). Blood samples were collected by cardiac puncture. Immediately after euthanasia, the urinary bladder and kidneys were exposed, and urine and kidney fragments were collected using sterile instruments. The small mammals were identified by external and cranial morphology, karyotyping, and DNA sequencing of cytochrome b gene (17, 18). Specimens were deposited in the mammal collection of the Laboratório de Biologia e Parasitologia de Mamíferos Reservatórios Silvestres, Instituto Oswaldo Cruz (tagged as LBCE), Rio de Janeiro, Brazil.

### Bacteriological Procedures

Individual urine samples were processed immediately in a mobile laboratory. Three to five drops of urine were inoculated to three different culture mediums: 5 mL of liquid EMJH medium (Difco, BD, Franklin Lakes, NJ, USA), 5 mL of semisolid Fletcher medium (Difco, BD, Franklin Lakes, NJ, USA), and 5 mL of liquid EMJH medium supplemented with an antibiotic cocktail named STAFF (19). Kidney fragment samples were macerated into EMJH using a 5 mL syringe. After inoculation, the tubes were maintained at room temperature and sent to the Reference Laboratory on Rio de Janeiro after 5 days due to Biosafety standards (4,000 km away from the initial collection site). There, tubes were incubated at 28°C and evaluated weekly for 4 months using dark-field optical microscopy. If contamination occurred, the liquid cultures were filtered or once again transferred to the EMJH and STAFF.

### Serology

To detect anti-*Leptospira* antibodies, we conducted the MAT according to World Organization for Animal Health standards (20). Strains related to 22 serogroups were used as antigens, using the highest titer obtained to identify the infecting serogroup. Animals were considered seroreactive when titration was ≥50, as carrier/host animals tend to present low titers (21).

### PCR

DNA was extracted from all individual kidney samples using the DNeasy® Blood & Tissue Kit (QIAamp, Qiagen, France) as recommended by the manufacturer. First, PCR was conducted using primers targeting a short region of the *lip*L32 gene (241 bp), reported to be present only in pathogenic leptospires (LipL32-45F: 5′-AAG CAT TAC CGC TTG TGG TG-3′ and LipL32-286R: 5′-GAA CTC CCA TTT CAG CGA TT-3′) (21). Second, *lip*L32 positive samples were subjected to a nested PCR targeting a partial region of *sec*Y gene. An initial reaction targeting a 549 bp region was conducted using the primers secY_outer_F (5′-ATGCCGATCATTTTTGCTTC-3′) and secY_outer_R (5′- CCGTCCCTTAATTTTAGACTTCTTC-3′). Finally, amplicons were included in a second reaction using the primers secY_inner_F (5′-CCTCAGACGATTATTCAATGGTTATC-3′) and secY_inner_R (5′- AGAAGAGAAGTTCCACCGAATG-3′) (Mathieu Picardeau, personal communication, November 27, 2019), providing an expected amplicon of 410 bp. In all reactions, for each set of samples, ultrapure water was used as negative control, while 10 fg of DNA extracted from *Leptospira interrogans* serovar Copenhageni (Fiocruz L1-130) was used as positive control. PCR products were analyzed by gel electrophoresis in 1.5–2% agarose and visualized under UV light, after GelRed® staining.

### Sequencing and Phylogenetic Analysis

The *sec*Y amplicons were directly sequenced using the Big Dye Terminator v. 3.1 Cycle Sequencing Ready Reaction Kit (Applied Biosystems) in a 3100 Automated DNA Sequencer according to the manufacturer's instructions. The nucleotide sequences were deposited in GenBank under accession numbers MT361666-MT361685. Phylogenetic analysis was accomplished using Pairwise/Blast/NCBI, SeqMan v. 7.0, ClustalW v. 1.35 (22) and BioEdit v. 7.0.1 (23) software for editing and sequence analysis. A maximum likelihood (ML) tree was constructed using the Tamura-Nei model with the gamma distribution (TN + G) in MEGA X software (24), as it was determined to be the best-fitting model of DNA substitution using the Bayesian information criterion. Information about sequences used for phylogenetic analysis is shown in the Supplementary Material.

### Statistics

We calculated the prevalence of infection by *Leptospira* sp., considering mammal carrier/host orders (rodents and marsupials), localities (FSC, RFH, FPZ, and FEC), forest strata (soil, understory, or canopy) and species. Prevalence of infection represents the proportion of PCR positive carrier/hosts by the total number of analyzed individuals.

Considering differences in conservation status among the study areas and differences among small mammal species in terms of their habits and habitat use, we tested the influence of carrier/host species, carrier/host orders, study area, and forest strata on the prevalence of infection by *Leptospira* spp. using generalized linear models (GLM). Best models were chosen using the corrected Akaike information criterion (AICc), where the suitable models presented Delta AICc ≤ 2. The GLM analysis followed a binomial distribution. The analyses were performed with R version 3.4.2 (25) using the vegan package (26). We also tested differences in *Leptospira* infection between two rodent families (Cricetidae and Echimyidae), with different evolutionary histories, using a chi-square test.

## Results

**Figure 2:**
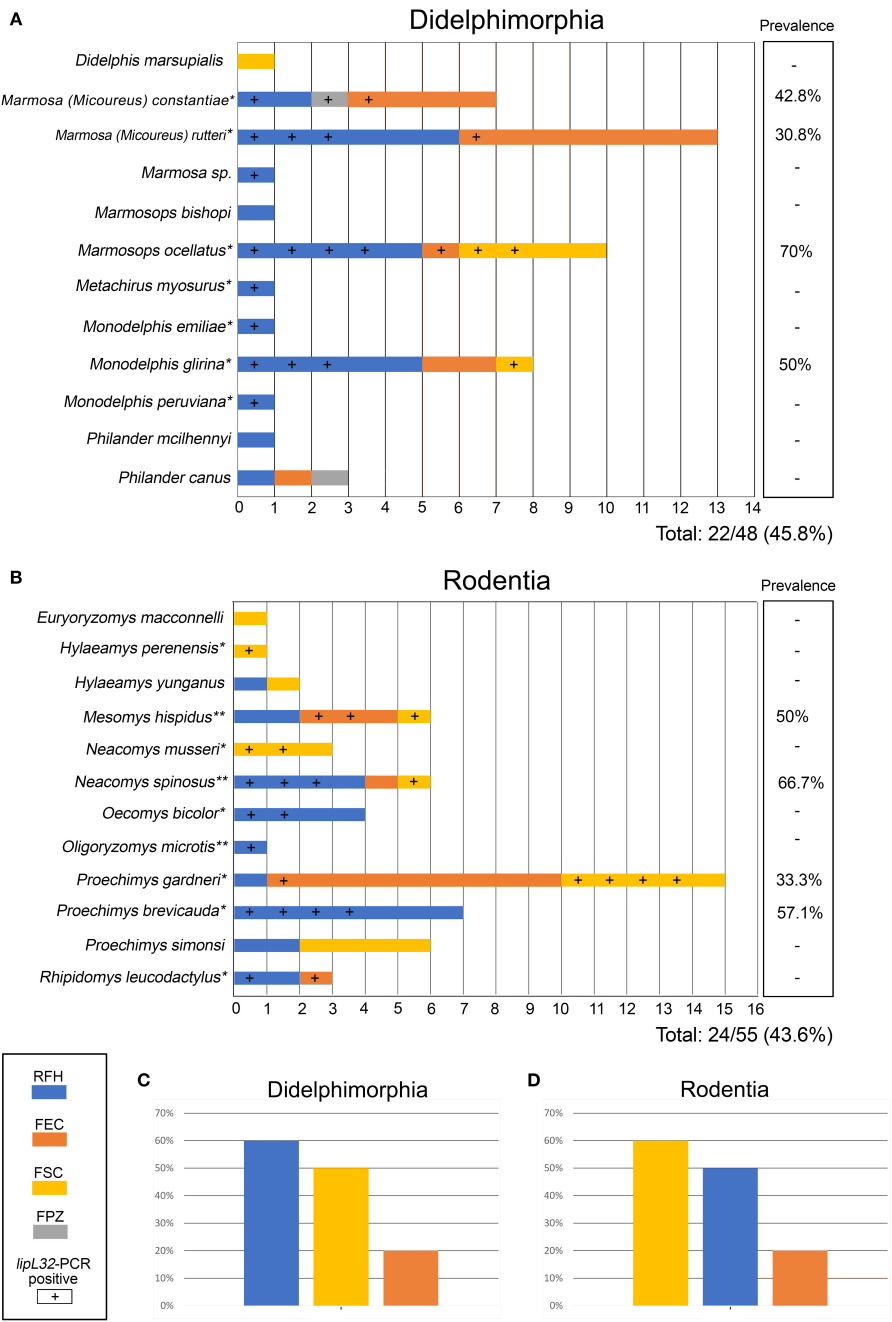
Small mammals (**A**: Didelphimorphia; **B**: Rodentia) included in the present study according to study area (shown by different colors as indicated in the figure). Samples positive for pathogenic *Leptospira*, detected by *lipL32* gene amplification, are indicated by a (+) sign. Columns at right show prevelance values for each host. Pathogenic *Leptospira* prevalence on Didelphimorphia **(C)** and Rodentia **(D)** of each collection site are also presented. *Species with first *Leptospira* DNA detection. **Species already described as carrier hosts of *Leptospira* by Bunnell at al. (29) and Cortez et al. (30). Species for which all samples were negative for Leptospira have no asterisk. Study areas located in the state of Acre: Floresta do Seringal Cachoeira (FSC), Reserva Florestal Humaitá (RFH), Floresta do Parque Zoobotânico (PPZ), and Floresta Experimental Catuaba (FEC).

We analyzed 103 animals, including 55 wild rodents and 48 marsupials, belonging to 23 species (Figures 2A,B). Of 15 serum samples subjected to MAT, only one (6.6%) was positive, a sample from the marsupial *Marmosa (Micoureus) rutteri* that was reactive to two *L. interrogans* serogroups (Australis and Autumnalis) at a titer of 1:100 each. All kidney culture attempts were negative. The most important result was the high prevalence (44.7%) of the *lip*L32 gene confirmed by PCR. Of the 15 sera submitted to MAT, six were *lip*L32 positive and nine were negative by PCR (including the only positive in MAT). We detected leptospiral DNA in kidney samples from carrier/hosts representing 16 species (*n* = 46/103, overall prevalence of 44.7%) in all four study areas investigated. Of the 23 mammalian carrier/host species studied, 16 had at least one leptospiral DNA-positive specimen (Figures 2A,B). In the carrier/host *Marmosops ocellatus, Leptospira* was found in 7/10 (70%) specimens, the highest prevalence in marsupials (Figure 2A). In rodents, *Neacomys spinosus* showed the highest percentage of *lipL32*-PCR positives (4/6, 66.7%) (Figure 2B).

The prevalence by study area was 12/23 (52.2%) in FSC, 26/49 (53.1%) in RFH, 1/2 (50%) in FPZ and 7/29 (24.1%) in FEC. Considering mammal orders, 22/48 (45.8%) marsupials and 24/55 (43.6%) rodents were PCR-positive. The prevalence by forest strata were 13/29 (44.8%) in canopy, 26/59 (44.1%) in soil and 6/14 (42.9%) in understory. The prevalences for each species are shown in Figures 2A,B, and prevalances according to collection site are show on Figure 2C (marsupials) and Figure 2D (rodents).

Considering only the PCR positive samples, 12/46 (26.1%) were positive in FSC, 26/46 (56.5%) in RFH, 1/46(2.2%) in FPZ, and 7/46 (15.2%) in FEC. Among mammal orders, 22/46 (47.8%) marsupials and 24/46 (52.2%) rodents were PCR positive. Also, considering forest strata, 13/45 (28.9%) in canopy, 26/45 (57.8%) in soil, and 6/45 (13.3%) in understory were PCR positive.

According to GLM analyses, the model with study area was suitable; however, the null model was also suitable, indicating no relation between the prevalence of infection by *Leptospira* sp. in small mammals considering the studied variables (**Table 2**). There were no differences in *Leptospira* infection between Cricetidae (12/21 = 57.1%) and Echimyidae (12/34 = 35.3%) (χ^2^ = 2.52, *p* = 0.1124, df = 1).

**Figure 3:**
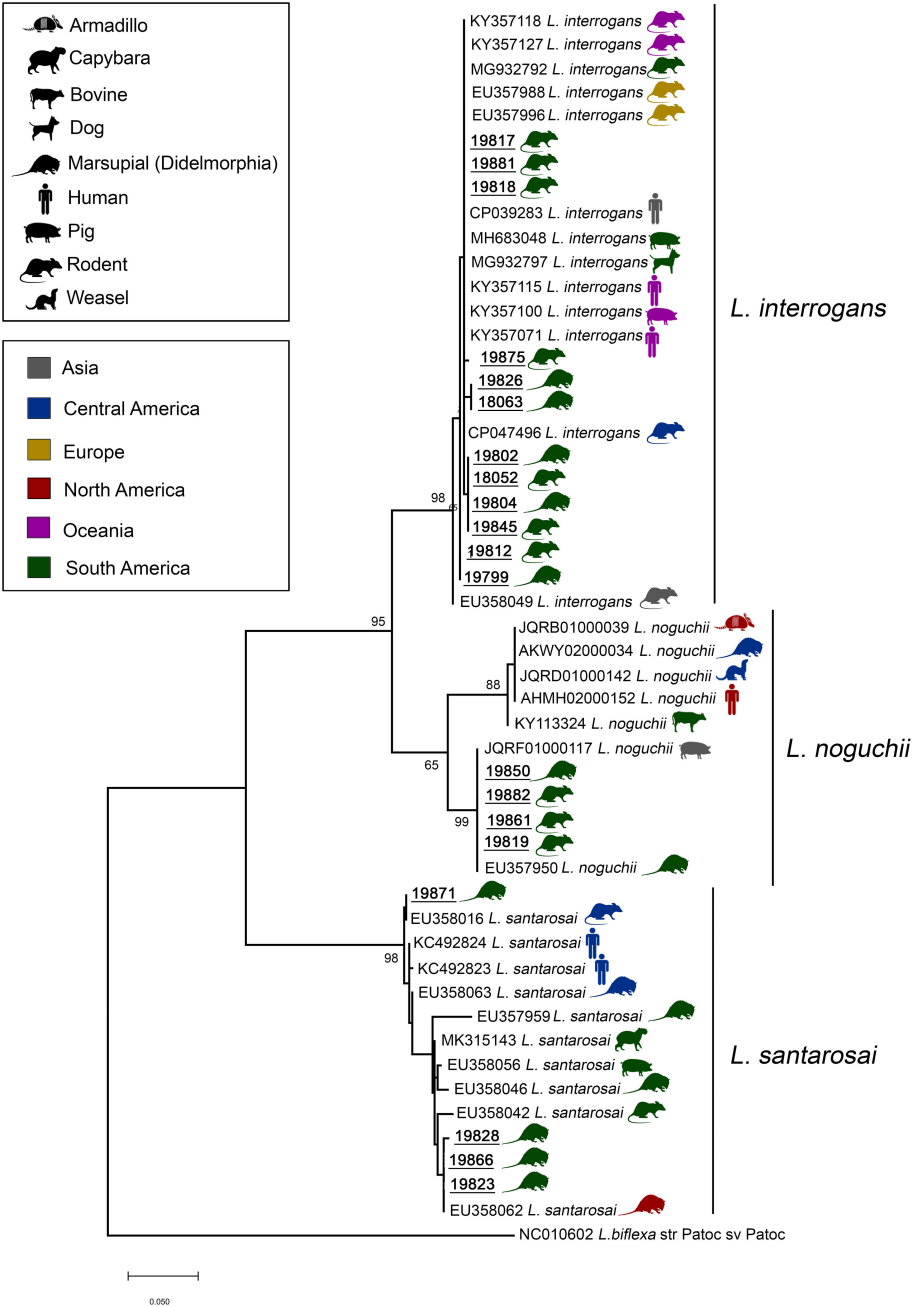
Maximum likelihood phylogenetic tree inferred from partial *sec*Y gene sequences (410 bp) of *L. interrogans, L. noguchii*, and *L. santarosai* from this study (bold and underlined) and GenBank sequences from other carrier/hosts (accession numbers are shown). Hosts are indicated by vectors and geographical locations by colors, as indicated in the figure. Numbers at nodes are bootstrap values >50%. *Leptospira biflexa* strain “Patoc” is the outgroup taxa.

DNA from 20 samples representing 13 carrier mammalian species was amplified and sequenced using the *sec*Y genetic marker (410 bp), providing a species-specific identification of leptospires. In three samples, it was not possible to perform phylogenetic analysis due to low sequence quality. Pairwise/Blast/NCBI comparisons with the GenBank *sec*Y gene dataset identified them as *L. interrogans* (*n* = 12), *L. noguchii* (*n* = 4) and *L. santarosai* (*n* = 4) (Table 1). Phylogenetic analysis based on ML TN+G tree including *sec*Y sequences from different carrier/hosts confirmed species identification (Figure 3). Sequence data included in phylogenetic analysis are shown in the Supplementary Material. Sequences of *L. interrogans* from the present study clustered together with sequences of *Leptospira* isolated from small mammals but also isolated from humans, swine, and dogs from different geographical locations (Figure 3) with high support value (98%). Two main *L. noguchii* clusters are observed, with all sequences from the present study grouping with high support value (99%) with sequences of *Leptospira* isolated from swine and opossum, from China and Peru, respectively, and distant from sequences from other carrier/hosts (Figure 3). A main cluster well-supported (98%) was observed with *L. santarosai* sequences from the present study and those from different carrier/hosts and geographical locations, including small mammals, humans, swine, and capybara, all from Americas (Figure 3).

**Table 1:** Pathogenic *Leptospira* genetically identified through secY gene sequencing in small mammals of the western Amazon.

**Carrier/Host**	***Leptospira***	**Collection**	**Sample ID**
	**species**	**Site**	**(LBCE)**
**DIDELPHIMORPHIA**			
*Marmosa (micoureus) constantiae*	*L. interrogans*	RFH	19802
	*L. noguchii*	FPZ	19850
*Marmosa (Micoureus) rutteri*	*L. interrogans*	RFH	19826
*Marmosops ocellatus*	*L. interrogans*	RFH	19804
	*L. interrogans*	FEC	18063
*Marmosops ocellatus*	*L. santarosai*	FSC	19866
*Metachirus myosurus*	*L. santarosai*	FSC	19828
*Monodelphis glirina*	*L. interrogans*	RFH	19799
	*L. santarosai*	FSC	19871
*Monodelphis peruviana*	*L. santarosai*	RFH	19823
**RODENTIA**			
*Mesomys hispidus*	*L. noguchii*	FSC	19861
*Neacomys spinosus*	*L. interrogans*	RFH	19818
	*L. interrogans*	RFH	19845
	*L. interrogans*	FSC	19875
*Neacomys musseri*	*L. interrogans*	FSC	19881
*Oligoryzomys microtis*	*L. interrogans*	RFH	19817
*Proechimys gardneri*	*L. noguchii*	FSC	19882
*Proechimys gardneri*	*L. interrogans*	FEC	18052
*Proechimys brevicauda*	*L. interrogans*	RFH	19812
*Rhipidomys leucodactylus*	*L. noguchii*	RFH	19819

*Study areas located in the state of Acre: FSC, Floresta do Seringal Cachoeira; RFH, Reserva Florestal Humaitá; PPZ, Floresta do Parque Zoobotânico; FEC, Floresta Experimental Catuaba*.

## Discussion

To our knowledge, this is the first study that investigated the leptospiral infection in small mammals in the Brazilian Western Amazon forest. Only one sample (6.6%), originating from the marsupial *Marmosa (Micoureus) rutteri*, was reactive against two *L. interrogans* serogroups (Australis and Autumnalis) with titers of 1:100 each. A limitation of this study was the small number of samples submitted to MAT, as obtaining sera from wild animals is not a trivial procedure. However, as reservoirs tend to show a seronegative response (27), the focus of this study was the identification of carriers/hosts by bacterial isolation and molecular techniques.

No bacterial isolate was obtained, possibly due to the expected low sensitivity of this technique. It is known that bacterial culturing of leptospires is fastidious, laborious, and difficult to perform (28). Additionally, the transport of samples from the field Laboratory on Acre State to the Reference Laboratory on Rio de Janeiro State delayed the filtration and reseeding of the samples, and consequently impaired bacterial isolation. Thus, the use of molecular techniques contributed for improving sensitivity of leptospiral detection, providing a broader understanding of the potential significance of infection.

We could identify, by PCR, seven Didelphimorphia and nine Rodentia species that could act as carriers of leptospires in the Amazon region. Our findings show, 13 new carrier/hosts (Figures 2A,B). Additionally, three species (*Mesomys hispidus, Neacomys spinosus* and *Oligoryzomys microtis*) were already described as leptospiral carriers in the Peruvian Amazon (29, 30) but described for the first time in the Brazilian region. Although the role of wild animals in the leptospirosis transmission cycle is unclear, the high diversity of carrier/host species found and the lack of correlation between a specific taxonomic level and a forest stratum with leptospiral infection suggest wide dissemination of the bacteria in those environments. Some studies found a higher occurrence of *Leptospira* infection in specific species of small mammals or a specific habitat/locality (9, 31). In our study, although we had observed differences in prevalence among study areas, the GLM model had no differences in relation to the null model (Table 2). Thus, there was no clear evidence of influence of those study areas with different land use and levels of conservation on the *Leptospira* prevalence.

**Table 2:** Generalized linear models (GLM) for the prevalence of infection by *Leptospira* sp. in small mammals from Western Amazon, Acre, Brazil.

**Models**	**AICc**	**Delta**	**Weight**	**K**	**Log-Likelihood**
Locality	141.5	0	0.374	4	−66.535
Null	142	0.54	0.285	1	−69.993

*AICc, corrected version of Akaike information criterion; Delta, Difference between the AICc value of a model and that of the best model; Weight, Akaike weights; K, number of parameters of the model*.

Samples from the present study clustered in highly supported clades with *L. interrogans, L. noguchii*, and *L. santarosai*. Interestingly, sequences from the present study were similar (>98%) to sequences identified in rodents and marsupials from different locations, including all continents (Figure 3). Moreover, while *L. noguchii* sequences were closest to each other, forming a distinct clade almost exclusively with sequences from South America. *L. interrogans* and *L. santarosai* sequences were closely related to sequences from other carrier/hosts from different geographical locations, including dogs and humans. This reinforces that these strains are widespread geographically and between carrier/host species.

From the epidemiological perspective, the identification of the animal species that may act as carriers/hosts is crucial since each species has a particular habitat use and geographic distribution. Thus, our data comprise 16 new mammalian species, described in Table 1, that can be carrier/hosts in the Brazilian Western Amazon (10). Those species were found in all study areas and in all habitat strata, showing that *Leptospira* could be widely distributed. All of the 23 mammal species have a known geographic distribution in the Amazon (32, 33). Considering the 16 PCR positive species, the most tolerant species are often found in peri-urban and rural forest fragments, and therefore, the most in contact with humans are the marsupials, *D. marsupialis* and *P. canus*, and rodent species *Proechimys gardneri* (32). Regarding other carrier/host species, besides their potential role in the *Leptospira* transmission in the Amazon region, some of them could also have an epidemiological importance in other Brazilian regions since they have a wider geographic distribution.

Moreover, genetic analysis showed that *Leptospira* species are circulating in different ecosystems including humans and other animals as carrier/hosts. This finding confirms the importance of a One Health context to study leptospirosis. Finally, this study reports the first evidence of the diversity of small mammals as leptospiral carriers/hosts in the western Brazilian Amazon forest.

## Data Availability Statement

All datasets generated for this study are included in the article/Supplementary Material.

## Ethics Statement

The animal study was reviewed and approved by The following licenses were used to conduct the study: permanent license to collect zoological material number 13373 (SISBIO–ICMBIO) and CEUA LW-39/14 license (Ethics Committee on animal use–FIOCRUZ).

## Author Contributions

LM paper writing, animal sampling, field work, PCR and MAT laboratory procedures, and project coordination. SB paper writing, bibliography research, field work, animal sampling, and data analysis. MD Leptospira sequencing, phylogenetic analysis, figures development, and paper writing. RP paper writing, bibliography research, and data analysis. NdA paper writing, field work, animal sampling, PCR procedures, and data analysis. PD'A paper writing, field work, animal sampling, animal identification by karyotyping, and DNA sequencing. AB paper writing, animal sampling, field work, animal identification by external, and cranial morphology. CC paper writing, field work, animal sampling, animal identification by external, and cranial morphology. AV PCR procedures, analysis, and paper writing. GM serological procedures, analysis, and paper writing. BT paper writing, animal identification by karyotyping, DNA sequencing, and statistical analysis. FC-C leptospira sequencing analysis and paper writing. WL project coordination and paper writing. All authors contributed to the article and approved the submitted version.

## Conflict of Interest

The authors declare that the research was conducted in the absence of any commercial or financial relationships that could be construed as a potential conflict of interest.
